# IL-23 plasma level is strongly associated with CMV status and reactivation of CMV in renal transplant recipients

**DOI:** 10.1186/s12865-016-0175-7

**Published:** 2016-10-03

**Authors:** Mahmoud Sadeghi, Imad Lahdou, Gerhard Opelz, Arianeb Mehrabi, Martin Zeier, Paul Schnitzler, Volker Daniel

**Affiliations:** 1 Department of General, Visceral and Transplant Surgery, University of Heidelberg, Im Neuenheimer Feld 110, D-69120 Heidelberg, Germany; 2 Transplantation Immunology, University of Heidelberg, Heidelberg, Germany; 3 Department of Nephrology, University of Heidelberg, Heidelberg, Germany; 4 Center for Infectious Diseases, Virology, University of Heidelberg, Heidelberg, Germany

**Keywords:** Kidney transplantation, CMV, IL-23, Th17, CMV-IgG

## Abstract

**Background:**

Cytomegalovirus seropositivity is an independent risk factor for atherosclerosis in patients with ESRD. Donor CMV seropositivity is associated with higher graft loss. Dendritic cells, macrophages and Th17 lymphocytes are defined as producers of IL-23. IL-23 is thought to be involved in the promotion of Th17 cell polarization. Latent CMV-induced Th17 might be involved in the pathogenesis of CMV infection in patients with ESRD. We aimed to evaluate associations of Th17-dependent cytokines with ESRD, CMV status and post-transplant outcome in kidney transplantation.

**Results:**

IL-21 plasma levels were similar in patients and healthy controls (*p* = 0.47), whereas IL-9 (*p* = 0.02) and IL-23 (*p* < 0.0001) levels were significantly higher in ESRD patients. CMV-seronegative (*p* = 0.002) and –seropositive (*p* < 0.001) patients had significantly higher IL-23 plasma levels than controls. CMV-seropositive patients showed excessively higher IL-23 (*p* < 0.001) plasma levels than CMV-seronegative patients. Patients with post-transplant CMV reactivation had higher IL-23 plasma levels than patients without CMV reactivation (*p* = 0.025).

**Conclusions:**

Our results indicate that latent CMV induces IL-23. IL-23 might be an inflammatory mediator of latent CMV infection in patients with ESRD and predisposes patients for post-transplant CMV reactivation.

## Background

End-stage renal diseases (ESRD) are associated with immunodeficiency and inflammation [1]. Previous studies reported on reduced numbers of circulating naive T cells, suggesting to indicate a reduction of Treg subpopulations as well [1]. Post-transplant immunologic responses are regulated by T helper (Th)1 or Th2 cells [2, 3]. The Th17 cells are a subset of effector- Th which specifically secretes IL-17. The cytokine IL-23 is produced by dendritic cells, macrophages, and Th17 pro-inflammatory cells [4, 5]. IL-23 is thought to be involved in the promotion of Th17 cell polarization. Th17 cells produce IL-17 and IL-22 and play an essential role in inflammatory diseases [5, 6]. Th2 cells were initially described as the main source of IL-9 [7]. Aside from them Th9, Th17 and peripherally induced T regulatory (iTreg) cells were also capable of producing IL-9 [7]. IL-9 is a plethoric cytokine and plays a role in allergy, inflammation, and cancer [7].

CMV infection is an unexpectedly high and independent risk factor for atherosclerosis in patients with ESRD, HIV+ patients and healthy individuals [8–12]. A recent study demonstrated a reduced rate of cardiovascular death after cytomegalovirus prophylaxis in renal transplant patients [13]. During the progression of renal failure in patients with chronic kidney disease, numbers of CMV-specific T cells increased [8]. Without CMV prophylaxis, donor and recipient CMV seropositivity are detrimental factors for long-term renal allograft survival and post-transplant CMV infection [14–16] whereas CMV prophylaxis prevented acute rejection (AR) and improved graft function [17]. It has been suggested that Th17 activation might contribute to the pathogenesis of latent CMV infection [18]. The association of Th17, IL-17 and Th17-dependent cytokines such as IL-21 with ESRD and transplantation outcome has been studied previously [18–26]. We aimed to evaluate the association of Th17-dependent cytokines in potential kidney transplant recipients with ESRD and CMV seropositivity and we tried to calculate the predictive value of these cytokines for post-transplant outcome.

## Methods

### Demographic data

The study was approved by the Ethical Committee of the University of Heidelberg. Pre-operative plasma levels of the cytokines IL-9, IL-21 and IL-23 were measured in 117 patients with ESRD (aged 49.8 ± 16.3 years, 54 female) who underwent kidney transplantation. Seventy patients from our center were followed for 1 year at the Heidelberg transplantation center and post-transplant events such as AR, DGF, graft loss and viral infections including CMV and BK-virus were analyzed. Recipient characteristics are shown in Table 1. ESRD was caused by glomerulonephritis, pyelonephritis, polycystic disease, diabetes or autoimmune diseases. Only one patient received preemptive kidney transplant. Mean duration of pre-transplant dialysis was 65.5 ± 51.5 months (rage: 2–304). There were 16 retransplant patients with non-significant different distribution in the 2 patient groups. The post-transplant administered immunosuppressive regimens were similar in the two groups (Table 1). Patients were classified as either CMV+ (*n* = 68) or CMV- (*n* = 49) by CMV serostatus. Post-transplant CMV pp65 antigenemia was determined and the presence of >3 detectable CMV pp65 positive cells in 500,000 peripheral leukocytes was defined as reactivation or symptomatic infection [27]. The CMV reactivation was confirmed by CMV-DNA detection in CMV pp65 antigen-positive patients. Routinely, all patients were monitored for CMV infection starting on day 10 post-transplant. Recipients who received kidneys from CMV-negative donors were evaluated for CMV pp65 antigen during the first 6 months post-transplant monthly and yearly thereafter. All recipients who obtained D+ kidneys received CMV prophylaxis with 900 mg daily valganciclovir for 3 months and were tested weekly during the first 3 months post-transplant, twice monthly for 4 to 6 months post-transplant, monthly for 7 to 12 months post-transplant, and 1 to 4 times yearly thereafter. The administration of CMV prophylaxis explains the relatively low percentage of patients with active CMV infection eligible for our study. Post-transplant BKV was determined by quantitative analysis of BKV DNA. More than 100 BKV copies/mL denotes as (re) activated infection. Twenty-seven healthy controls with no known active infectious and other inflammatory diseases served as controls to establish references for the studied cytokines.

**Table 1:** IL-9, IL-21 and IL-23 plasma levels in healthy controls and ESRD patients with and without CMV seropositivity

Parameters	Controls (*n* = 27)	ESRD (*n* = 117)	*p*
IL-9 (pg/ml)	799 ± 2203	435 ± 930	0.74
IL-21 (pg/ml)	368 ± 751	194 ± 213	0.61
IL-23 (pg/ml)	2.9 ± 11	10 ± 17	<0.001
	Controls (*n* = 27)	CMV-IgG- (*n* = 49)	
IL-9 (pg/ml)	799 ± 2203	336 ± 810	0.53
IL-21 (pg/ml)	368 ± 751	156 ± 175	0.25
IL-23 (pg/ml)	2.9 ± 11	5.4 ± 7.8	0.002
	Controls (*n* = 27)	CMV-IgG+ (*n* = 68)	
IL-9 (pg/ml)	799 ± 2203	506 ± 1007	0.31
IL-21 (pg/ml)	368 ± 751	221 ± 235	0.96
IL-23 (pg/ml)	2.9 ± 11	14 ± 20	<0.0001
	CMV-IgG- (*n* = 49)	CMV-IgG+ (*n* = 68)	
IL-9 (pg/ml)	336 ± 810	506 ± 1007	0.02
IL-21 (pg/ml)	156 ± 175	221 ± 235	0.12
IL-23 (pg/ml)	5.4 ± 7.8	14 ± 20	<0.001

Mann-Whitney-*U* test was used to calculate p values

Data are given as mean ± SD

### Determination of CMV activation

#### DNA detection and PCR amplification

CMV DNA was extracted as previously reported [28]. Briefly CMV DNA was extracted from 200 μl EDTA blood samples and purified using the QIAamp blood kit (QIAGEN, Hilden, Germany). For amplification of CMV IE-1 gene DNA, a nested PCR consisting of two successive sets of 35 cycles was performed in a Gene Amp PCR System 2400 thermocycler (Perkin Elmer, Norwalk, CT). All DNA extraction and amplification reactions carried appropriate parallel negative controls to detect contamination at any stage in the procedure. PCR products were electrophoresed in a 2 % agarose gel containing 0.5 mg/ml ethidium bromide and visualized under ultraviolet illumination.

### CMV pp65 antigen detection

CMV pp65 antigen was detected as previously reported [29]. Briefly, about 8 mL of EDTA blood was used for isolation of leukocytes and 500,000 leukocytes were spun carefully on a slide using a cytospin centrifuge. Cells were fixed and stained with an anti-CMV pp65 mouse monoclonal antibody, washed, and further incubated with an anti-mouse-immunoglobulin G FITC-labeled antibody. More than 3 positive leukocytes per 500,000 cells denote an activated CMV infection [27].

### Detection of active BKV infection by real-time PCR

BKV infection was detected as previously reported [30]. Briefly, Nucleic acid was isolated from untreated plasma using the QIAamp blood kit (Qiagen; Hilden; Germany) according to the manufacturer’s instructions. For quantitative analysis of BKV DNA, 5 μL of extracted nucleic acids was amplified as described previously [30]. More than 100 BKV copies/mL denote an activated infection [31].

### Sample collection and determination of plasma IL-9, IL-21 and IL-23

All samples were collected immediately before transplantation. Within 2 h after the blood was drawn plasma was separated from cells by centrifugation at 1550 × g for 10 min. The plasma was snap frozen and stored at -30 °C until testing. The plasma levels of IL-9, IL-21 and IL-23 were measured with a commercial test developed by Komabiotech South Korea using ELISA kits. Captured antibodies were added to previously coated plates with cytokine antibodies and incubated. Biotinylated detection antibody was added to each test well and incubated. HRP-conjugated streptavidin was added to each well. Then color development enzyme was added to each well and after 15 min the results were obtained by measuring the absorbance reading. The protocol provided by the assay manufacturer was strictly followed and all samples were tested undiluted according to the instructions of the manufacturer. Each sample was tested in duplicate and the mean of each sample was analyzed.

### Statistical analyses

Categorical and continuous variables were analyzed using chi square, Fisher exact and Mann-Whitney-U tests. The most sensitive cut-off values were calculated by ROC curve analysis. Uni- and multivariable logistic regression analyses were applied to identify risk factors for CMV (re) activation. All statistical analyses were performed with the Statistical Package for the Social Sciences (SPSS, 18.0; SPSS Inc., Chicago, IL, USA). After Bonferroni correction, *p* values <0.05 were defined as statistically significant.

## Results

### IL-9, IL-21 and IL-23 levels in healthy controls and patients with ESRD

**Fig. 1:**
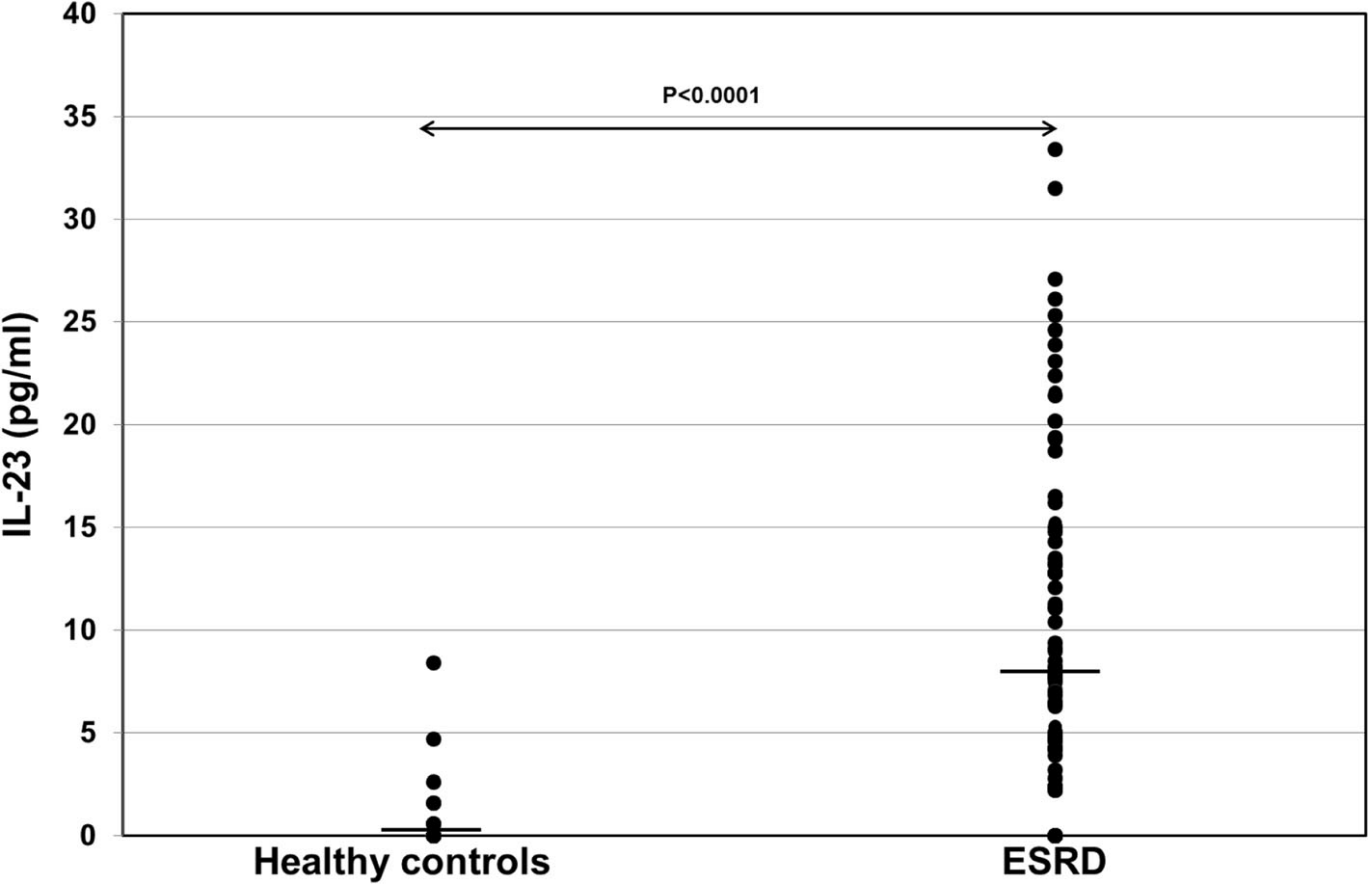
IL-23 plasma levels in patients with ESRD (*n* = 117) and healthy controls (*n* = 27). Plasma IL-23 levels were determined retrospectively using ELISA kits and data were statistically analyzed using Mann-Whitney-*U* test. Horizontal bars show median values in each study group. ESRD patients had significantly higher IL-23 levels pre-transplant than healthy controls

Plasma levels of IL-9 (*p* = 0.74) and IL-21 (*p* = 0.61) were similar in patients with ESRD and healthy controls, whereas the plasma level of IL-23 (*p* < 0.001) was strongly increased in patients with ESRD (Fig. 1 and Table 1).

### IL-9, IL-21 and IL-23 levels in first transplant and retransplant recipients, in patients with hemodialysis and peritoneal dialysis and in male and female patients with ESRD

Plasma levels of IL-9, IL-21 and IL-23 were similar in first and retransplant recipients (*n* = 101 and *n* = 16, respectively), hemodialysis (*n* = 99) and peritoneal dialysis (*n* = 17) as well as in male and female patients (*p* = n.s.; data not shown). Although CMV-seropositive patients had significantly longer pre-transplant dialysis time, there were no correlations between pre-transplant dialysis time (IL-9: *p* = 0.78, IL-21: *p* = 0.97 and IL-23: *p* = 0.96, respectively), serum Cr (*p* = 0.68, *p* = 0.36 and *p* = 0.73, respectively), recipient’s age (*p* = 0.28, *p* = 0.11 and *p* = 0.33, respectively) and IL-9, IL-21 and IL-23 levels.

### IL-9, IL-21 and IL-23 levels in patients with and without CMV seropositivity

**Fig. 2:**
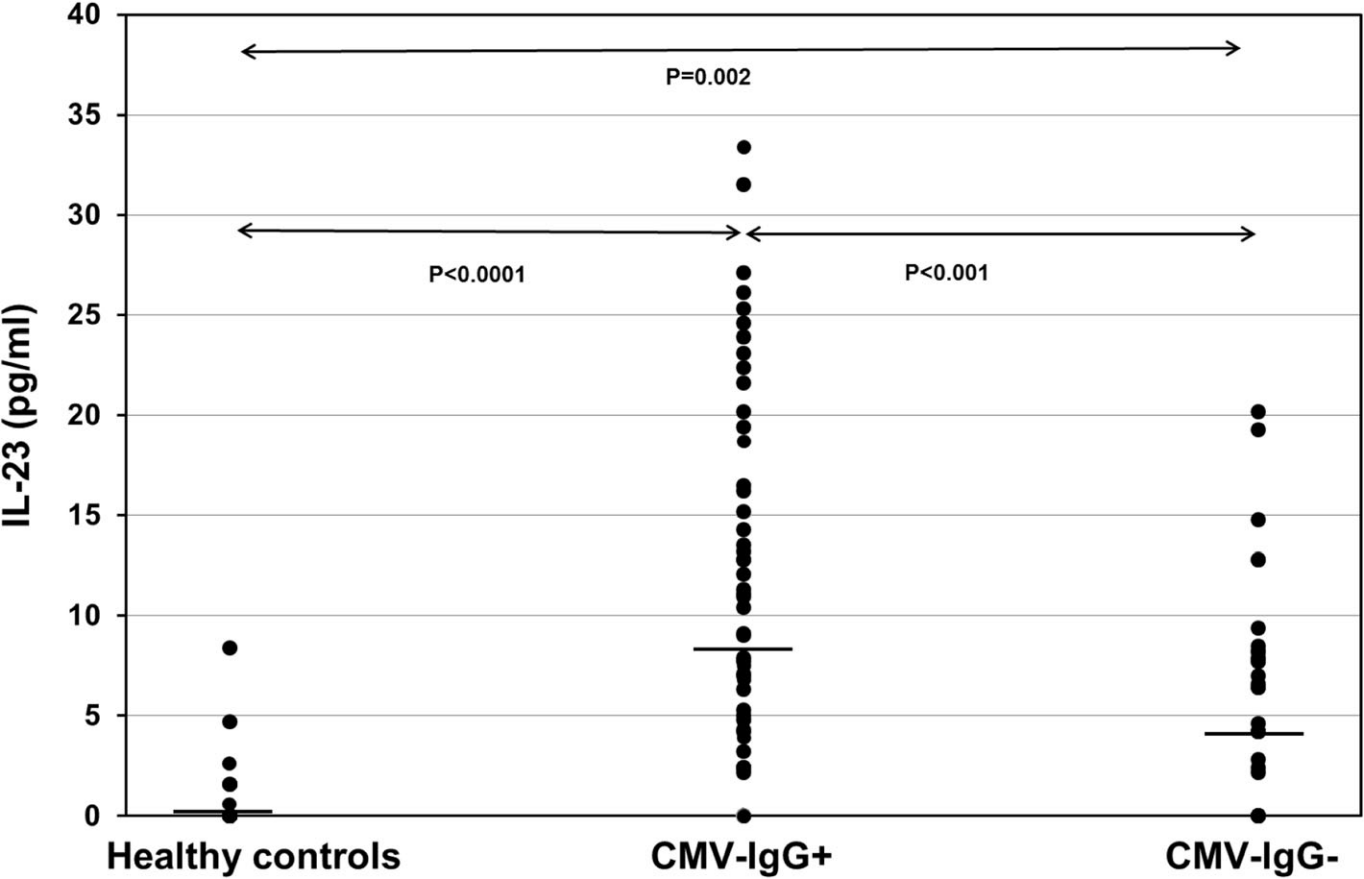
IL-23 plasma levels in ESDR patients with CMV-IgG status and healthy controls. Plasma IL-23 levels were determined retrospectively using ELISA kits and data were statistically analyzed using Mann-Whitney-*U* test. Horizontal bars show median values in each study group. CMV-IgG+ ESDR patients (*n* = 69) had significantly higher IL-23 levels pre-transplant than CMV-IgG- ESDR patients (*n* = 48) and healthy controls (*n* = 27)

**Fig. 3:**
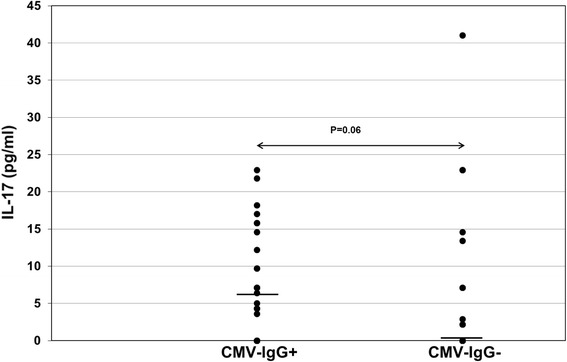
IL-17 plasma levels in ESDR patients with CMV-IgG status. Plasma IL-17 levels were determined retrospectively using ELISA kits and data were statistically analyzed using Mann-Whitney-*U* test. Horizontal bars show median values in each study group. CMV-IgG+ ESDR patients (*n* = 28) had significantly higher IL-17 levels pre-transplant than CMV-IgG- ESDR patients (*n* = 25). In 25 plasma samples IL-17 was not detectable. Sixteen of 25 CMV-IgG- and 9 of 28 CMV-IgG+ patients had undetectable IL-17 (*p* = 0.02) and CMV-IgG+ patients showed a trend of higher IL-17 plasma levels than patients without CMV-IgG suggesting that Th17 cells were activated

Interestingly, CMV-seronegative (*p* = 0.002) as well as CMV-seropositive (*p* < 0.001) patients showed higher IL-23 plasma levels than healthy controls (Fig. 2 and Table 1). Furthermore, CMV-seropositive patients showed significantly higher IL-23 plasma levels than CMV-seronegative recipients (*p* < 0.001) (Table 1). IL-9 and IL-21 plasma levels in ESRD, CMV-seronegative and CMV-seropositive patients were similar to those in healthy individuals (*p* = n.s.). There was a trend of higher IL-9 plasma levels in CMV-seropositive compared with CMV-seronegative patients (*p* = 0.02) whereas IL-21 was similar in both patient groups (*p* = 0.12) (Table 1). IL-17 levels were measured in 53 plasma samples. In 25 plasma samples IL-17 was not detectable. Sixteen of 25 CMV-IgG- and 9 of 28 CMV-IgG+ patients had undetectable IL-17 (*p* = 0.02) and CMV-IgG+ patients showed a trend of higher IL-17 plasma levels than patients without CMV-IgG (7.5 ± 7.4 pg/ml vs 5.2 ± 10.0: *p* = 0.06) suggesting that Th17 cells were activated (Fig. 3).

### IL-9, IL-21 and IL-23 levels in patients with and without post-transplant events

**Fig. 4:**
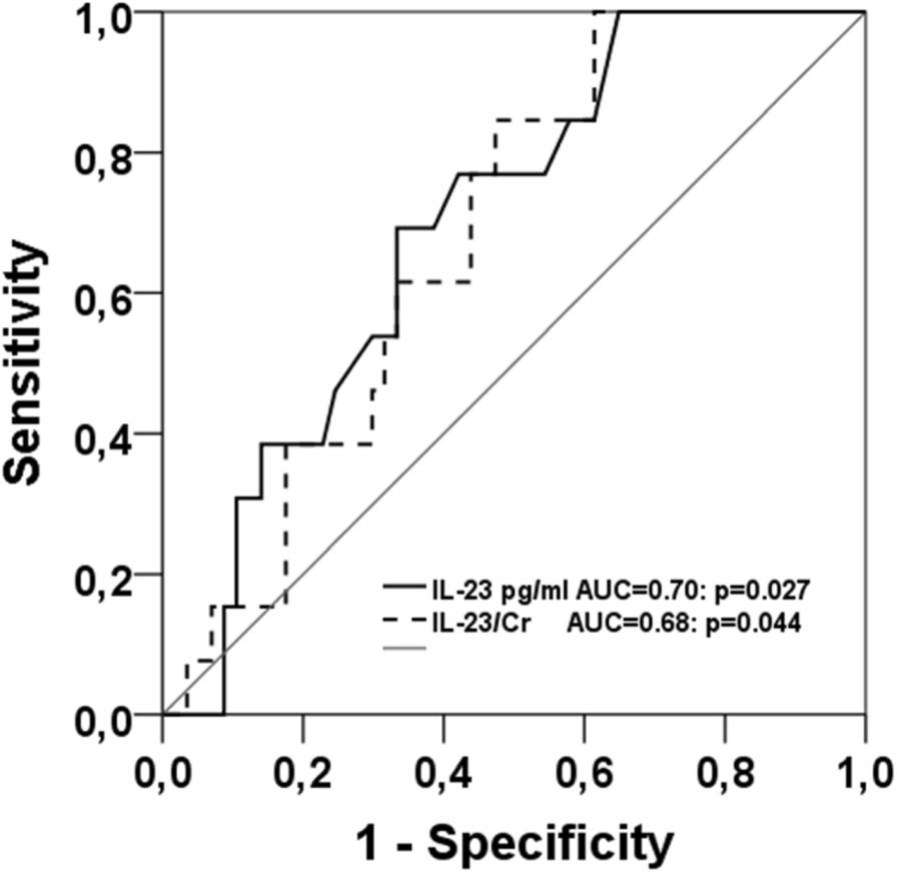
Pre-transplant IL-23 plasma levels in kidney transplant recipients with and without post-transplant CMV infection. Plasma IL-23 levels were analyzed retrospectively using ELISA kits and data were statistically analyzed using Mann-Whitney-*U* test. Horizontal bars show median values in each study group. Patients with post-transplant CMV infection (*n* = 13) had significantly higher IL-23 levels pre-transplant than patients without CMV infection (*n* = 57) and healthy controls (*n* = 27)

**Fig. 5:**
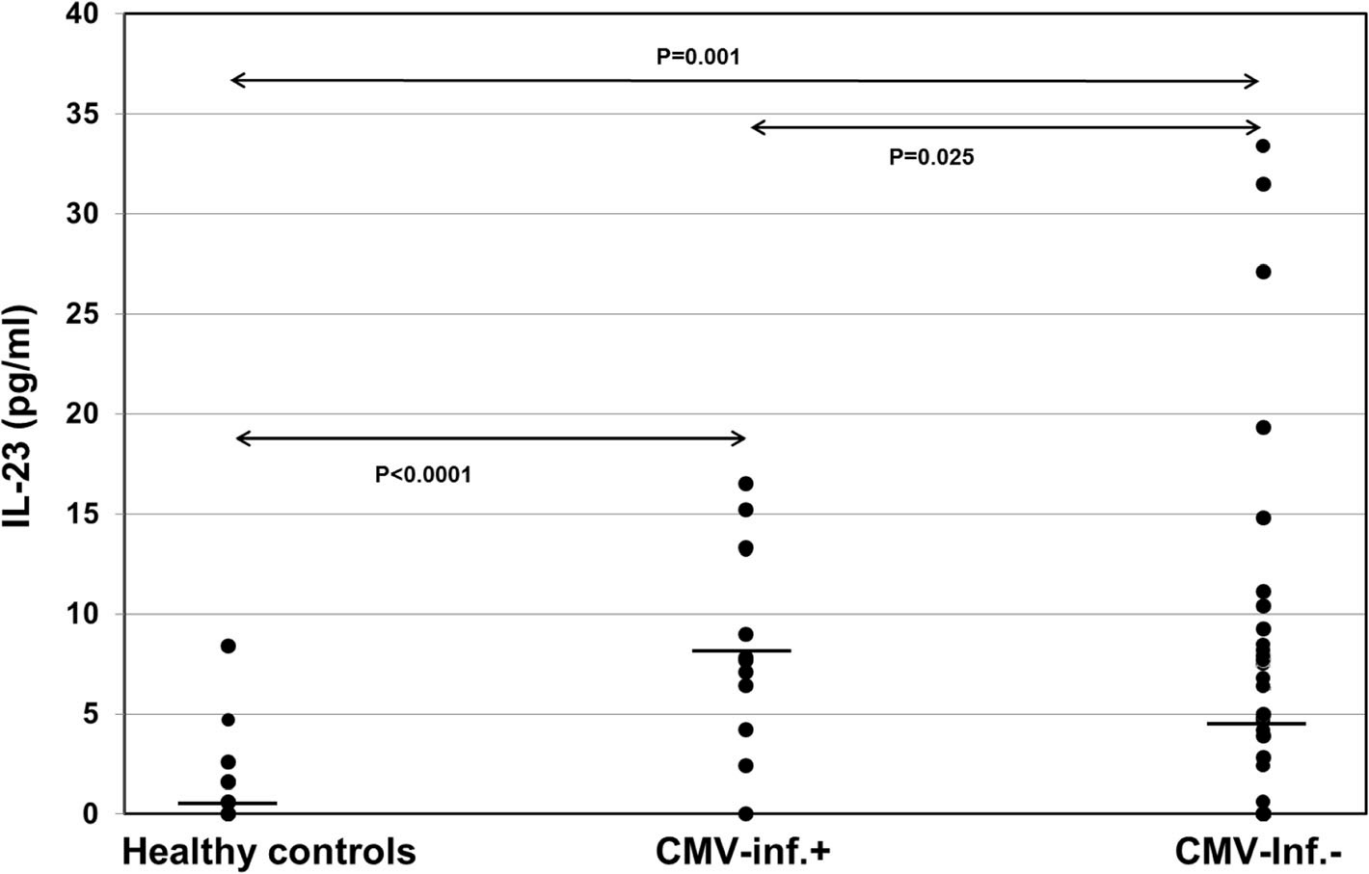
ROC curve analysis for the calculation of diagnostic accuracy, cut-off value, sensitivity and specificity of pre-transplant IL-23 plasma levels to predict post-transplant CMV reactivation in patients. At a cut-off value for IL-23 of ≥7 pg/ml ROC curve analysis showed a sensitivity of 69 % and a specificity of 67 %, a PPV of 31 % and a NPV of 89 %

Seventy patients were followed for 1 year post-transplant. Eight patients experienced AR (Banff classification 2007; Ia, IIb and III), 27 patients borderline rejection (BR) and 20 patients DGF. Six patients had biopsy-proven ATN. Plasma levels of IL-9, IL-21 and IL-23 were similar in patients with and without post-transplant AR, BR, patients with and without rejection (AR + BR), ATN or DGF (Table 2). Twenty-four patients showed evidence of viral infection, thereof BK-virus infection in 11, CMV infection in 7 and CMV plus BK-virus infection in 6 patients. Only one patient had biopsy-proven BKV nephropathy. Median value of BKV was 30×10^7^ copies/ml plasma (range: 17×10^4^−58×10^9^). Median duration of BKV viremia was 173 days (range: 19–365 days). CMV-seronegative recipients (R-) who received grafts from CMV-seronegative donors (D-) (D-/R-; *n* = 14) did not experience CMV infection. In contrast, 4 of 21 (19 %) D+/R-, 6 of 14 (43 %) D-/R+ and 3 of 22 (14 %) D+/R+ transplants developed CMV infection/reactivation including hepatitis, GI-bleeding, viremia, thrombocytopenia, pneumonia, colitis, esophagitis with antigenemia (*n* = 13; 239 ± 379 pp65 antigen+/500,000 leucocytes, range 10–1138) and viremia (*n* = 8; 449240 ± 521917 CMV-DNA copies/ml). Ten of 13 patients with CMV (re) infection had higher IL-23 than median and 3 of 13 lower than median value (*p* = 0.02). Patients with CMV or CMV plus BKV infection had similar plasma levels of cytokines as patients with BK infection only (*p* = n.s.; data not shown) suggesting that CMV as well as BKV infections increase IL-9, IL-21, and IL-23 cytokine levels. However, patients with post-transplant CMV disease (*n* = 13; 150 ± 106 days post-transplant, range 41–363 days) had higher pre-transplant plasma levels of IL-23 (8.6 ± 4.4 vs 8.0 ± 17: *p* = 0.025) and IL-23/Cr ratios (*p* = 0.040) than patients without CMV disease after transplantation (*n* = 57), suggesting an association of high IL-23 plasma levels with CMV antigenemia and symptomatic CMV (re) infection post-transplant (Fig. 4). Plasma levels of IL-9 (*p* = 0.80), IL-21 (*p* = 0.39), IL-9/Cr (*p* = 0.67) and IL-21/Cr (*p* = 0.34) were not statistically different between the two groups. Regression analysis indicated that a pre-transplant IL-23 plasma level of >7 pg/ml is a risk factor for post-transplant CMV infection/reactivation and symptomatic infection (RR = 4.50, confidence interval 1.23–16.52: *p* = 0.023). ROC curve analysis with respect to post-transplant CMV disease showed a sensitivity of 69 % and a specificity of 67 % at a cut-off value for IL-23 of ≥7 pg/ml (*p* = 0.025) (Fig. 5).

**Table 2:** IL-9, IL-21 and IL-23 plasma levels in patients with and without post-transplant events

Patients	IL-9	IL-21	IL-23
	*p*	*p*	*p*
AR+ (*n* = 8) vs AR- (*n* = 109)	0.43	0.84	0.54
BR+ (*n* = 27) vs BR- (*n* = 90)	0.71	0.93	0.13
AR + BR+ (*n* = 35) vs AR- (*n* = 82)	0.44	0.85	0.28
DGF+ (*n* = 20) vs DGF- (*n* = 97)	0.95	0.42	0.46
ATN+ (*n* = 6) vs DGF- (*n* = 111)	0.60	0.60	0.07

Mann-Whitney-*U* test was used to calculate p values

Data are given as mean ± SD

## Discussion

Post-transplant CMV reactivation and active infection continues to be a major risk factor for transplant recipients [32]. CMV (re) infection is one of the most frequent causes of morbidity and mortality in transplant recipients early and late post-transplant [33, 34]. It was reported that CMV infection is an independent risk factor for atherosclerosis and A-V fistula in hemodialysis patients [8–10, 35, 36]. Thus, there is a need to identify pre-transplant patients at risk of developing CMV disease post-transplant in spite of the administration of CMV prophylaxis.

Our data show that pre-transplant IL-23 plasma levels >7 pg/ml are associated with a high risk of developing CMV disease during the first year post-transplant. High IL-23 plasma levels indicate a strong activation of Th17 lymphocytes, dendritic cells and macrophages. Th17 lymphocytes might be involved in the elimination of virus-infected cells and might represent a sensitive tool for identifying patients with replicating virus [18, 37]. A high proportion of IL-17 positive patients and a trend of higher IL-17 plasma levels in our CMV-IgG positive patients is in agreement with previous results. Part of the ESRD patients harbor virus whose replication rate is low and inhibited by the patient’s immune system (latent infection). However, when these patients receive immunosuppression post-transplant, the treatment-impaired immune system is unable to suppress virus replication and the patient develops CMV disease, associated with early death or atherosclerosis as long-term consequence [38] The use of valaciclovir, valganciclovir or ganciclovir for 3 to 6 months is an effective prophylaxis of cytomegalovirus disease in high risk patients. However, CMV prophylaxis does not prevent CMV disease in every recipient of a CMV-seropositive transplant. Our data show that CMV-seropositive as well as CMV-seronegative recipients who had received organs from seropositive donors are at high risk of post-transplant CMV reactivation despite CMV prophylaxis. Pre-transplant IL-23 measurement as a surrogate marker might identify patients with ongoing anti-CMV responses that might lead to developing CMV disease post-transplant. Post-transplant IL-23 monitoring as surrogate marker might indicate strong CMV replication despite CMV prophylaxis, suggesting the need of additional treatment options to cure impending CMV disease. Because Th17 lymphocytes might be involved in the elimination of virus-infected cells, IL-23 levels might reflect the potential of the immune system during immunosuppressive therapy to eliminate virus-producing cells. It appears from our data that the Th17 activation in patients with CMV disease was not strong enough to inhibit CMV replication. Therefore, it should be considered whether patients with excessively high IL-23 should receive more individualized immunosuppressive treatment in order to prevent over-immunosuppression and active virus disease. Similar to high-risk bone marrow transplant recipients, kidney transplant recipients with pre-transplant high IL-23 might be candidates of immunomodulatory therapy against CMV infection, including intravenous immune globulin and cytomegalovirus hyperimmune globulin as well as adoptive transfer of CMV-specific T-cells and donor/recipient vaccination strategies [39]. Further comparative studies of patients with and without high IL-23 are needed to confirm our results and our conclusion [40–43].

## Conclusion

Our data suggest that CMV-seropositive patients with high pre-transplant IL-23 should preferentially receive a CMV-seronegative graft in order to lower the risk of post-transplant CMV disease. IL-23 monitoring pre- and post-transplant might enable decisions concerning treatment options with the aim to decrease the risk of the post-transplant complications such as CMV disease and one might speculate that CMV prophylaxis in renal transplant recipients might reduce the rate of cardiovascular death.

## Abbreviations

AR: Acute rejection
ATN: Acute tubular necrosis
BR: Borderline rejection
CMV: Cytomegalovirus
DGF: Delayed graft function
EDTA: Ethylene diamine tetraacetic acid
ELISA: Enzyme-linked immunosorbent assay
ESRD: End-stage renal diseases
FITC: Fluorescein isothiocyanate
IgG: Immunoglobulin G
IL: Interleukin
PCR: Polymerase chain reaction
ROC: Receiver operating characteristic
Th: T helper cell
TNF: Tumor necrosis factor
Treg: T regulatory

## References

[1] Edozie FC, Nova-Lamperti EA, Povoleri GA, Scotta C, John S, Lombardi G (2014). Regulatory T-cell therapy in the induction of transplant tolerance: the issue of subpopulations. Transplantation.

[2] Nakagiri T, Inoue M, Minami M, Shintani Y, Okumura M (2012). Immunology mini-review: the basics of T(H)17 and interleukin-6 in transplantation. Transplant Proc.

[3] Daniel V, Sadeghi M, Wang H, Opelz G (2013). CD4+ CD25+ Foxp3+ IFNgamma + CD178+ human induced Treg (iTreg) contribute to suppression of alloresponses by apoptosis of responder cells. Hum Immunol.

[4] Shabgah AG, Fattahi E, Shahneh FZ (2014). Interleukin-17 in human inflammatory diseases. Postepy dermatologii i alergologii.

[5] Cheng LS, Liu Y, Jiang W (2015). Restoring homeostasis of CD4(+) T cells in hepatitis-B-virus-related liver fibrosis. World J Gastroenterol.

[6] Qu N, Xu M, Mizoguchi I, Furusawa J, Kaneko K, Watanabe K (2013). Pivotal roles of T-helper 17-related cytokines, IL-17, IL-22, and IL-23, in inflammatory diseases. Clin Dev Immunol.

[7] Schmitt E, Klein M, Bopp T (2014). Th9 cells, new players in adaptive immunity. Trends Immunol.

[8] Betjes MG, Huisman M, Weimar W, Litjens NH (2008). Expansion of cytolytic CD4 + CD28- T cells in end-stage renal disease. Kidney Int.

[9] Betjes MG, de Wit EE, Weimar W, Litjens NH (2010). Circulating pro-inflammatory CD4posCD28null T cells are independently associated with cardiovascular disease in ESRD patients. Nephrol Dial Transplant.

[10] Heybar H, Alavi SM, Farashahi Nejad M, Latifi M (2015). Cytomegalovirus infection and atherosclerosis in candidate of coronary artery bypass graft. Jundishapur J Microbiol.

[11] Goulenok T, Boyd A, Larsen M, Fastenackels S, Boccara F, Meynard JL (2015). Increased carotid intima-media thickness is not associated with T-cell activation nor with cytomegalovirus in HIV-infected never-smoker patients. Aids.

[12] Lee YL, Liu CE, Cho WL, Kuo CL, Cheng WL, Huang CS (2014). Presence of cytomegalovirus DNA in leucocytes is associated with increased oxidative stress and subclinical atherosclerosis in healthy adults. Biomarkers.

[13] Opelz G, Dohler B (2015). Reduced rate of cardiovascular death after cytomegalovirus prophylaxis in renal transplant recipients. Transplantation.

[14] Fitzgerald JT, Gallay B, Taranto SE, McVicar JP, Troppmann C, Chen X (2004). Pretransplant recipient cytomegalovirus seropositivity and hemodialysis are associated with decreased renal allograft and patient survival. Transplantation.

[15] Malaise J, Ricart MJ, Moreno A, Crespo M, Fernandez-Cruz L, Van Ophem D (2005). Cytomegalovirus infection in simultaneous pancreas-kidney transplantation. Transplant Proc.

[16] Petersen P, Schneeberger H, Schleibner S, Illner WD, Hofmann GO, Land W (1994). Positive donor and negative recipient cytomegalovirus status is a detrimental factor for long-term renal allograft survival. Transpl Int.

[17] Opelz G, Dohler B, Ruhenstroth A (2004). Cytomegalovirus prophylaxis and graft outcome in solid organ transplantation: a collaborative transplant study report. Am J Transplant Off J Am Soc Transplant Am Soc Transplant Surg.

[18] Tan YF, Yu SJ, Wang J, Li SJ (2012). Role of Treg/Th17 balance in the pathogenesis of cytomegalovirus infection. Xi Bao Yu Fen Zi Mian Yi Xue Za Zhi.

[19] Carvalho A, Cunha C, Di Ianni M, Pitzurra L, Aloisi T, Falzetti F (2010). Prognostic significance of genetic variants in the IL-23/Th17 pathway for the outcome of T cell-depleted allogeneic stem cell transplantation. Bone Marrow Transplant.

[20] Chung BH, Kim KW, Sun IO, Choi SR, Park HS, Jeon EJ (2012). Increased interleukin-17 producing effector memory T cells in the end-stage renal disease patients. Immunol Lett.

[21] Deteix C, Attuil-Audenis V, Duthey A, Patey N, McGregor B, Dubois V (2010). Intragraft Th17 infiltrate promotes lymphoid neogenesis and hastens clinical chronic rejection. J Immunol.

[22] Kim YG, Kim EY, Ihm CG, Lee TW, Lee SH, Jeong KH (2012). Gene polymorphisms of interleukin-17 and interleukin-17 receptor are associated with end-stage kidney disease. Am J Nephrol.

[23] Romanowski M, Kloda K, Osekowska B, Domanski L, Pawlik A, Safranow K (2015). Influence of the IL17A and IL17F gene polymorphisms on the long-term kidney allograft function and return to dialysis after kidney transplantation. Clin Transpl.

[24] San Segundo D, Lopez-Hoyos M, Fernandez-Fresnedo G, Benito MJ, Ruiz JC, Benito A (2008). T(H)17 versus Treg cells in renal transplant candidates: effect of a previous transplant. Transplant Proc.

[25] Matignon M, Aissat A, Canoui-Poitrine F, Grondin C, Pilon C, Desvaux D (2015). Th-17 Alloimmune Responses in Renal Allograft Biopsies From Recipients of Kidney Transplants Using Extended Criteria Donors During Acute T Cell-Mediated Rejection. Am J Transplant Off J Am Soc Transplant Am Soc Transplant Surg.

[26] Safinia N, Afzali B, Atalar K, Lombardi G, Lechler RI (2010). T-cell alloimmunity and chronic allograft dysfunction. Kidney Int Suppl.

[27] Asadullah K, Prosch S, Audring H, Buttnerova I, Volk HD, Sterry W (1999). A high prevalence of cytomegalovirus antigenaemia in patients with moderate to severe chronic plaque psoriasis: an association with systemic tumour necrosis factor alpha overexpression. Br J Dermatol.

[28] Sadeghi M, Daniel V, Naujokat C, Schnitzler P, Schmidt J, Mehrabi A (2008). Dysregulated cytokine responses during cytomegalovirus infection in renal transplant recipients. Transplantation.

[29] Sadeghi M, Lahdou I, Daniel V, Schnitzler P, Fusch G, Schefold JC (2012). Strong association of phenylalanine and tryptophan metabolites with activated cytomegalovirus infection in kidney transplant recipients. Hum Immunol.

[30] Sadeghi M, Daniel V, Schnitzler P, Lahdou I, Naujokat C, Zeier M (2009). Urinary proinflammatory cytokine response in renal transplant recipients with polyomavirus BK viruria. Transplantation.

[31] Bechert CJ, Schnadig VJ, Payne DA, Dong J (2010). Monitoring of BK viral load in renal allograft recipients by real-time PCR assays. Am J Clin Pathol.

[32] Eid AJ, Razonable RR (2010). New developments in the management of cytomegalovirus infection after solid organ transplantation. Drugs.

[33] Browne BJ, Young JA, Dunn TB, Matas AJ (2010). The impact of cytomegalovirus infection >/=1 year after primary renal transplantation. Clin Transpl.

[34] Heurlin N, Brattstrom C, Tyden G, Ehrnst A, Andersson J (1989). Cytomegalovirus the predominant cause of pneumonia in renal transplant patients. A two-year study of pneumonia in renal transplant recipients with evaluation of fiberoptic bronchoscopy. Scand J Infect Dis.

[35] Grandaliano G, Teutonico A, Allegretti A, Losappio R, Mancini A, Gesualdo L (2003). The role of hyperparathyroidism, erythropoietin therapy, and CMV infection in the failure of arteriovenous fistula in hemodialysis. Kidney Int.

[36] MacManiman JD, Meuser A, Botto S, Smith PP, Liu F, Jarvis MA (2014). Human cytomegalovirus-encoded pUL7 is a novel CEACAM1-like molecule responsible for promotion of angiogenesis. MBio.

[37] Camargo JF, Resende MR, Zamel R, Klement W, Bhimji A, Huibner S (2015). Potential role of CC chemokine receptor 6 in prediction of late-onset cytomegalovirus infection following solid organ transplant. Clin Transpl.

[38] Ducloux D, Courivaud C, Bamoulid J, Crepin T, Chalopin JM, Tiberghien P (2014). Polyclonal antithymocyte globulin and cardiovascular disease in kidney transplant recipients. J Am Soc Nephrol.

[39] Boeckh M, Fries B, Nichols WG (2004). Recent advances in the prevention of CMV infection and disease after hematopoietic stem cell transplantation. Pediatr Transplant.

[40] Abbas A, Gregersen I, Holm S, Daissormont I, Bjerkeli V, Krohg-Sorensen K (2015). Interleukin 23 Levels Are Increased in Carotid Atherosclerosis: Possible Role for the Interleukin 23/Interleukin 17 Axis. Stroke.

[41] Kave M, Shadman M, Alizadeh A, Samadi M (2015). Analysis of the association between IL-23R rs11209026 polymorphism and incidence of atherosclerosis. Int J Immunogenet.

[42] Zhang M, Cai ZR, Zhang B, Cai X, Li W, Guo Z (2014). Functional polymorphisms in interleukin-23 receptor and susceptibility to coronary artery disease. DNA Cell Biol.

[43] Courivaud C, Bamoulid J, Chalopin JM, Gaiffe E, Tiberghien P, Saas P (2013). Cytomegalovirus exposure and cardiovascular disease in kidney transplant recipients. J Infect Dis.

